# Prognostic factors on periapical surgery: A systematic review

**DOI:** 10.4317/medoral.20613

**Published:** 2015-10-09

**Authors:** Mireia Serrano-Giménez, Alba Sánchez-Torres, Cosme Gay-Escoda

**Affiliations:** 1 Dentistry Student, School of Dentistry, University of Barcelona, Spain; 2 DDS. Fellow of the Master of Oral Surgery and Orofacial Implantology. School of Dentistry of the University of Barcelona, Spain; 3 MD, DDS, MS, PhD, EBOS, Chairman and Professor of the Oral and Maxillofacial Surgery Department, School of Dentistry, University of Barcelona. Director of Master’s Degree Program in Oral Surgery and Implantology (EHFRE International University/FUCSO). Coordinator/Researcher of the IDIBELL Institute. Head of Oral and Maxillofacial Surgery and Implantology Department of the Teknon Medical Center, Barcelona, Spain

## Abstract

**Background:**

Analyze the most important prognostic factors when performing periapical surgery and compare the success rates of distinct authors.

**Introduction:**

Periapical surgery is an approach to treat non-healing periapical lesions and it should be viewed as an extension of endodontic treatment and not as a separate entity.

**Material and Methods:**

A search of articles published in Cochrane, PubMed (MEDLINE) and Scopus was conducted with the key words “prognostic factors”, “prognosis”, “periapical surgery”, “endodontic surgery” and “surgical endodontic treatment”. The inclusion criteria were articles including at least 10 patients, published in English, for the last 10 years. The exclusion criteria were nonhuman studies and case reports.

**Results:**

33 articles were selected from 321 initially found. Ten articles from 33 were excluded and finally the systematic review included 23 articles: 1 metaanalysis, 1 systematic review, 2 randomized clinical trials, 6 reviews, 12 prospective studies and 1 retrospective study. They were stratified according to their level of scientific evidence using the SORT criteria.

**Conclusions:**

Factors associated with a better outcome of periapical surgery are patients ≤45 years old, upper anterior or premolar teeth, ≤10 sized lesions, non cystic lesions, absence of preoperative signs and symptoms, lesions without periodontal involvement, teeth with an adequate root-filling length, MTA as root-end filling material, uniradicular teeth, absence of perforating lesions, apical resection < 3 mm, teeth not associated to an oroantral fistula and teeth with only one periapical surgery.

**Key words:**Prognostic factors, prognosis, periapical surgery, endodontic surgery and surgical endodontic treatment.

## Introduction

Endodontic treatment is usually performed in teeth with periapical lesions. However, in some cases the pathology persists. Thus, periapical surgery has to be perfomed. It is considered to be the last treatment option before the extraction of a tooth. The main objective of periapical surgery is to seal the root canal system, thereby enabling healing by forming a barrier between the irritants within the confines of the afected root and the periapical tissue. The success of periapical surgery is usually determined by both radiological signs and clinical signs and symptoms (1,2).

The indications for periapical surgery, based on the protocol proposed by the Spanish Society of Oral Surgery (3-5) are: 1) periapical disease affecting a permanent tooth subjected to endodontic treatment (of good quality), with pain and inflammation; 2) periapical pathology with prosthodontic or conservative restoration proven to be difficult to remove; 3) a radiotransparent lesion measuring over 8 to 10 mm in diameter; 4) symptomatic gutta-percha overfilling, or presence of a foreign body not amenable to orthograde removal (eg, fractured file); 5) other indications (patient requiring endodontic treatment and periapical surgery in a single session, fracture of the apical third, etc.).

As proposed by Lieblich (6) periapical surgery has to be performed in a tooth with no evidence of fracture and with an adequate periodontal status (less than 25% of vertical bone loss and periodontal pockets < 5 mm). Furthermore, the tooth must retain sufficient coronary structure for prosthesis and the patient should be able to tolerate the surgery. After 3 months of surgery, if the tooth remains symptomatic surgical retreatment in expert hands or extraction has to be performed, depending on each particular case. In absence of clinical signs and symptoms, the clinician can proceed to finish the coronal restoration.

There is scarce information regarding prognostic factors in periapical surgery. Most studies evaluate the results with respect to the retrograde filling material used. However, only a few studies such as a metaanalysis made by von Arx *et al*. (7) have evaluated other prognostic factors such as age, gender, type of tooth and the presence of a root pole.

The aim of this systematic review is to analyze the most important prognostic factors when performing periapical surgery and compare the success rates of distinct authors.

## Material and Methods

A search in Cochrane, PubMed (MEDLINE) and Scopus database was conducted (May 2014 to November 2014) with the key words “prognostic factors”, “prognosis”, “periapical surgery”, “endodontic surgery” and “surgical endodontic treatment”. Next, the terms were merged using the Boolean operator “AND”, in order to obtain the articles that included two or more of the used search terms.

The inclusion criteria were human prospective clinical studies about periapical surgery including at least 10 patients, published in English and 10 years aged articles. The exclusion criteria were non human studies and case reports because they constitute a low level of evidence.

## Results

Out of the 321 articles retrieved in the initial search, 288 were excluded due to the lack of data and/or lack of direct relationship with the subject. The full text of the remaining 33 was analyzed and finally, 23 articles with relevance were selected to be included in this systematic review: 1 metaanalysis, 1 systematic review, 2 randomized clinical trials, 6 reviews, 12 prospective studies and 1 retrospective study. Specifically, 12 of them have been subjected to the data extraction, synthesis and analysis of the data to perform a complete analysis about prognostic factors in periapical surgery (1).

**Figure 1:**
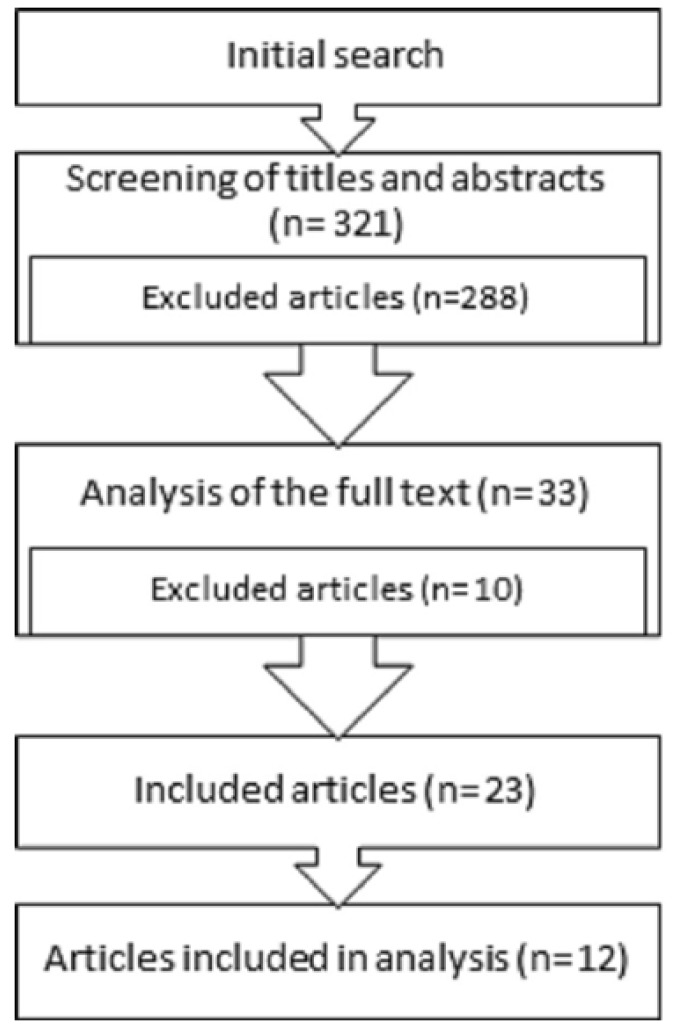
Flow of articles through the systematic review.

A summary which synthesizes the characteristics of each study has been made ( Table 1).

**Table 1:**
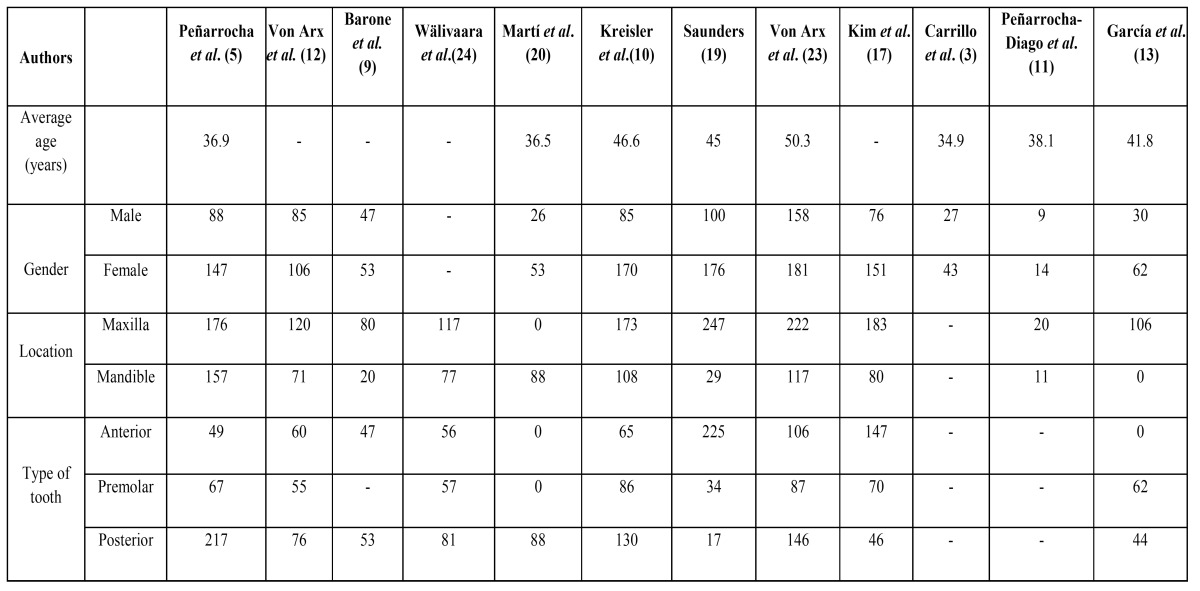
Distribution of teeth, gender and average age of participants in the prospective studies.

The SORT (Strength of Recommendation Taxonomy) criteria (8) were used to stratify the articles according to their level of evidence, as observed in  Table 2 Table 3 and  Table 4.

**Table 2:**
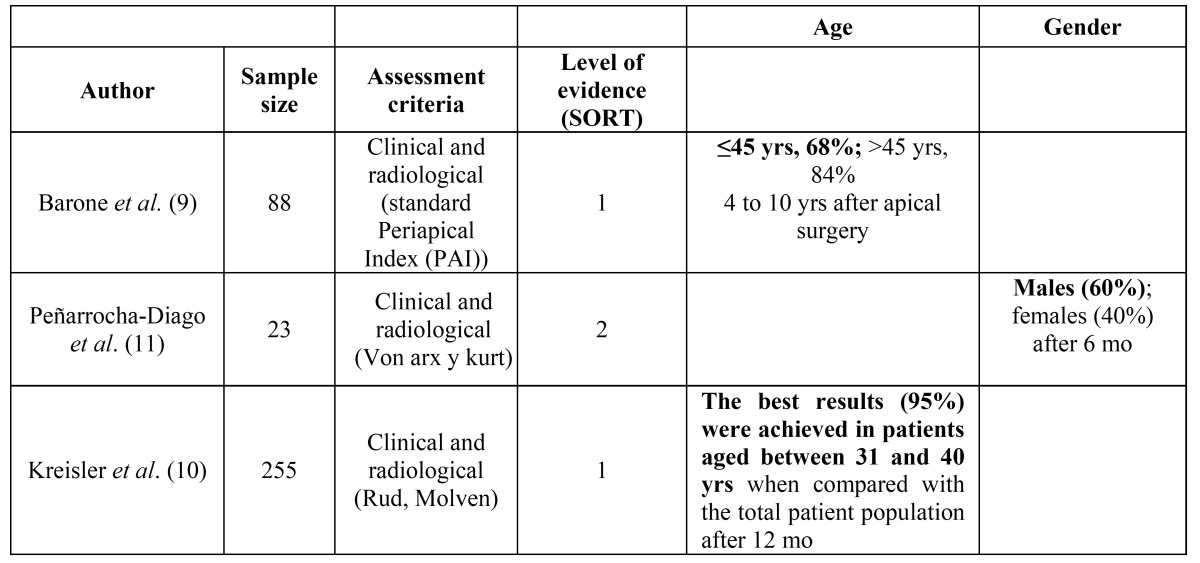
Success rate found for patient-related factors in the prospective studies used in this systematic review. The factors that positively affect the outcome of treatment are highlighted in bold.

**Table 3:**
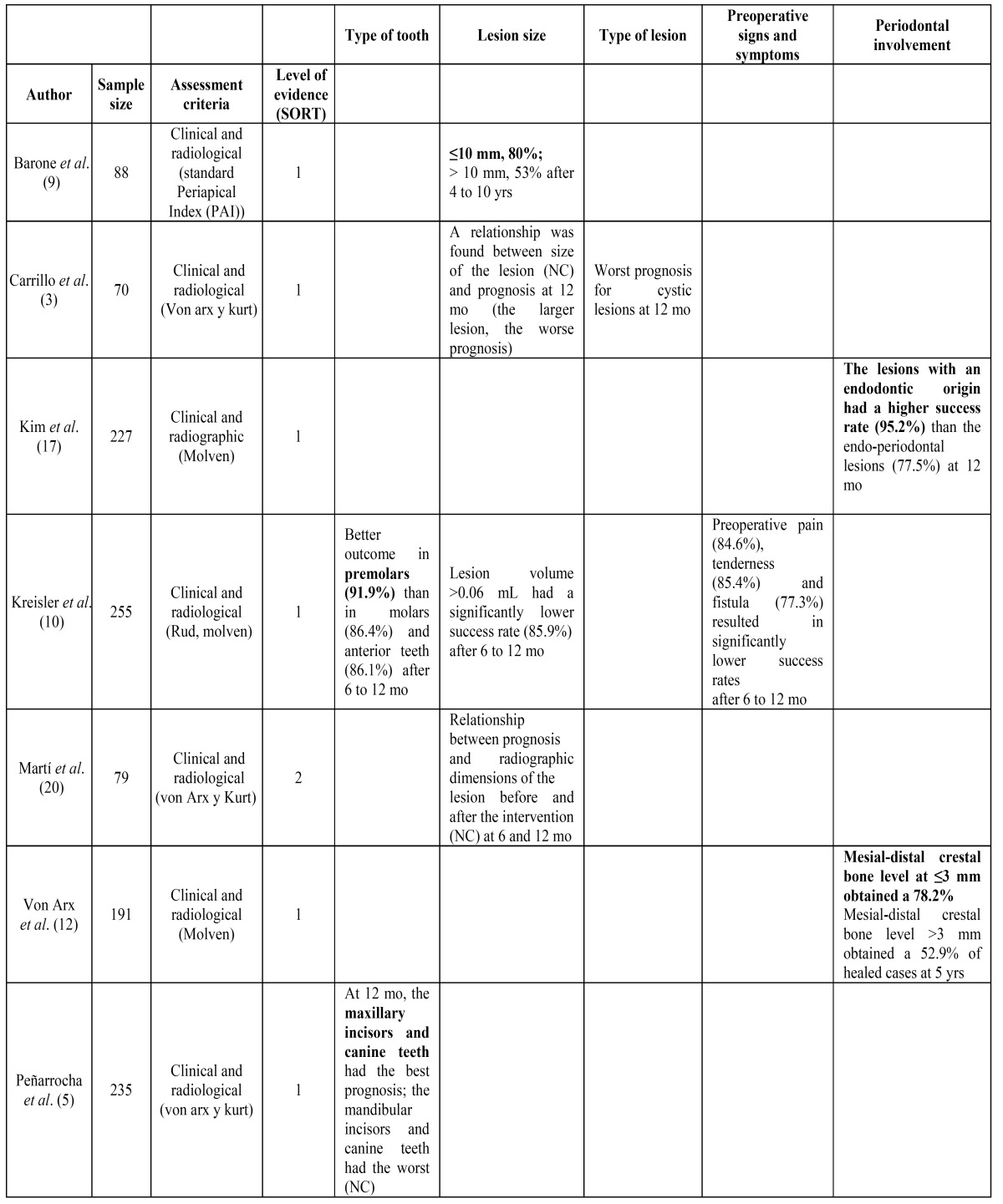
Success rate found for tooth-related factors in the prospective studies used in this systematic review. The factors that positively affect the outcome of treatment are highlighted in bold. NC: not cited.

**Table 4:**
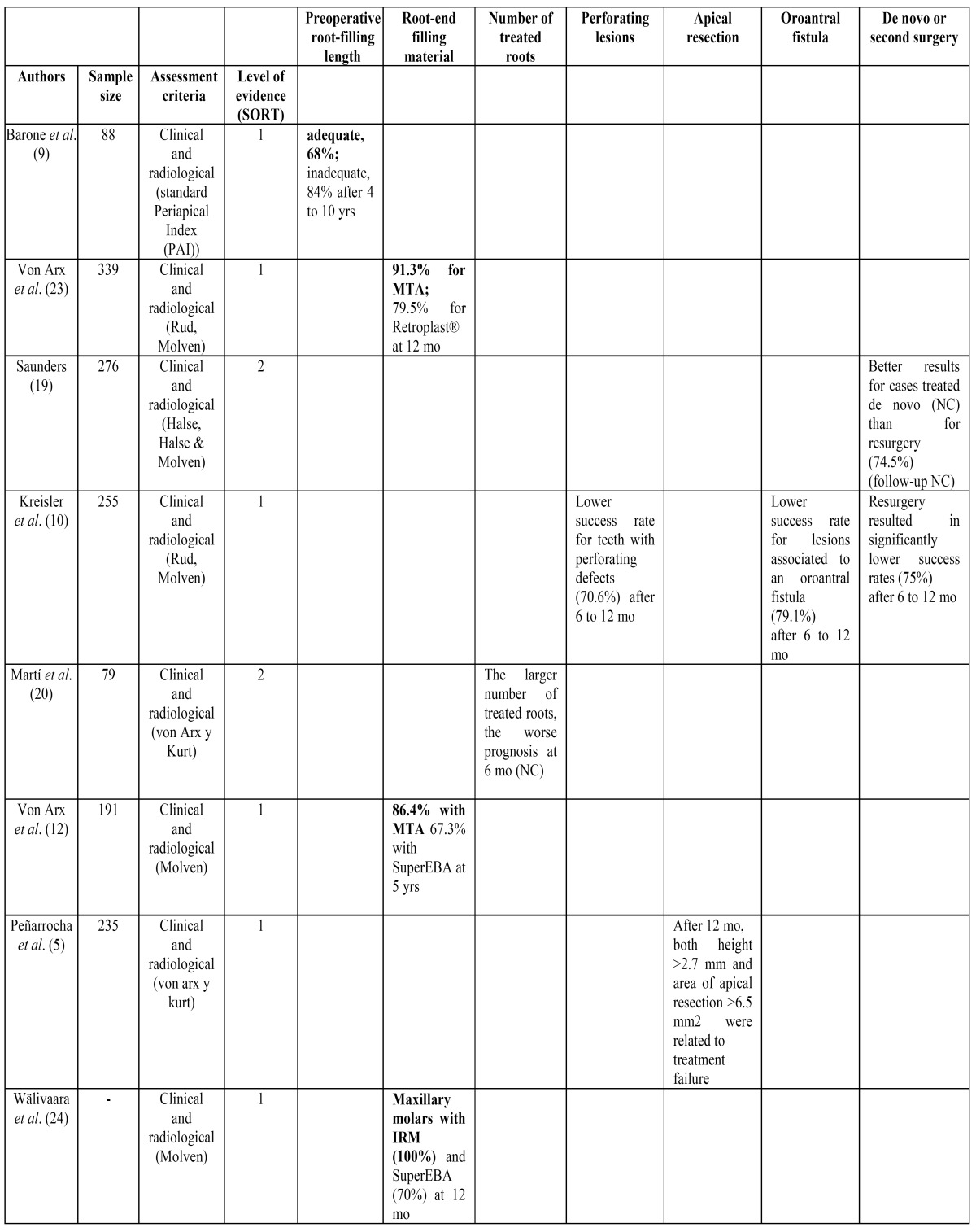
Success rate found for treatment-related factors in the prospective studies used in this systematic review. The factors that positively affect the outcome of treatment are highlighted in bold. NC: not cited.

The most important factors that affect the prognosis of periapical surgery have been divided in patient-related factors ( Table 2), tooth-related factors ( Table 3) and treatment-related factors ( Table 4) in order to a better comprehension of the results from this systematic review.

The assessment of methodological quality shows that from the 12 prospective studies, 9 are neither randomized nor blinded [3,8,9-11,13,15,17,18], 8 have an attrition bias due to loss of participants along the study [8,10,11,13,15,17,18,21] and 1 study presents a noticeable difference between the number of participants of each group [18]. Because of the heterogeneity of the available studies, it has not been possible to perform a statistical analysis.

## Discussion

Periapical surgery has always been considered as the last option prior to tooth extraction, with an unpredictable outcome. Today this technique has evolved so that we can discuss the periapical microsurgery, obtaining very good results and predictable healing of many periapical lesions associated with endodontic pulp pathology.

Von Arx *et al*. (7) published a literature review to clarify which are the most influential factors for the success of periapical surgery. They divided the studied factors in patient-related, tooth-related and treatment-related factors.

Regarding the patient-related factors, age and gender constitute the studied items in the literature. Only 2 studies (9,10) have found age to be an outcome predictor. Barone *et al*. (9) found a healing rate in patients older than 45 years of 84%, compared with 68% for younger patients. On the contrary, Kreisler *et al*. (10) obtained the best results (95%) in patients aged between 31 and 40 years of age, compared with the total population. However, definitive conclusions can not be drawn as most articles do not provide reliable or significant data on the importance of age. Likewise, gender seems not to be an outcome-related factor because only one study (11) found a statistically significant difference at 6 months between males and females, which had a success rate of 60% and 40%, respectively.

Regarding tooth-related factors, lesion size is one of the most studied. The largest lesions are associated with a worst prognosis. The study with the longest follow-up was that from Barone *et al*. (9) in which they found that lesions ≤ 10 mm had an 80% of success rate while the larger ones showed a success rate of 53% after a period from 4 to 10 years. A favorable prognosis of periapical surgery seems almost assured when the periapical lesion is less than 5 mm (5,7,12-14).

The relation between prognosis and type of tooth is not clear yet. Many authors agree that incisors and canines have higher success rates in endodontic surgery (5-7,9,12,14,15). This may be explained because the anterior sectors are more accessible and permit an excellent view of the operative field, thus obtaining a better apical seal. One of the two studies (5) selected for the analysis found a better prognosis in maxillary incisors and canine while the other one (10) obtained a greater success rate for premolars and a worst one for anterior teeth. Comparing the results of periapical surgery in upper and lower teeth, no differences have been found (15).

In a study made by Carrillo *et al*. (3) a significant worst prognosis was found for cysts after 12 months, although the authors do not mention the success rate percentage.

Teeth with periapical lesions having a concomitant endoperiodontal problem have a very low success rate (15,16). A study made by Kim *et al*. (17) found that endodontic origined lesions had a 95.2% success rate while endoperiodontal origined lesions had serious problems for healing, obtaining a success rate of 77.5% at 12 months after periapical surgery. Besides, the fact that the teeth do not show mesial or distal bone loss increases the healing rate. This seems to be due to the risk of short and long-term apical reinfection by bacteria moving toward the apex (12). A study made by von Arx *et al*. (12) showed that a mesial-distal crestal bone level less than 3 mm was a positive prognostic factor because a 78.2% successfully healed cases were obtained after 5 years. Contrarily, a lower success percentage of 52.9% was obtained in teeth with more than 3 mm from the cementoenamel junction.

The results of periapical surgery are influenced by the presence of pain both pre and postoperatively. A meta-analysis of von Arx *et al*. (7) claim that the preoperative pain results in a smaller success rate. This is in agreement with a multi center study (10) that found significant lower success rates for cases with preoperative pain, tenderness and presence of a fistula after 6 to 12 months. On the contrary, other studies do not find statistically significant differences in success rates in relation to the presence of preoperative pain (12,15,18).

Finally, regarding treatment-related factors, the outcome of periapical surgery can be severely compromised in teeth with poorly treated canals, so it is essential to perform an endodontic retreatment by an orthograde approach prior to the surgical treatment. On the other hand, the surgical retreatment has not shown better results compared to the initial surgical treatment (10,19). If the first time the periapical surgery is performed the treatment fails, it is recommended reoperation with micro surgical techniques that have shown great progress in recent years obtaining high success rates (15).

When the quality of the previous root canal treatment is inadequate worse results are found (6,7,12) compared to cases in which the length of the root canal filling is adequate. The length of the root canal treatment influences the success rate of periapical surgery (9,12,15). If the length of the root canal filling is short, the unfilled portion of the root canal may be the major source of infection and the material extruded out of the apex could favor the persistence of the lesion. When these areas are removed during surgical maneuvers, periapical surgery has good results even in teeth with root canals with inadequate working length (9,15). Besides, the larger number of treated roots, the worse prognosis of periapical surgery (20).

Many articles agree that the absence of an intracanal post in the tooth represents a plus for successful surgery (6,9,12,15). If present, the removal of the post could create cracks or fissures in the root inducing a split, which will result in the loosening of the tooth (21). Likewise, one study demonstrated a significantly lower success rate for teeth with perforating defects compared to teeth without them (10).

Teeth with a good coronal restoration are more likely to complete healing after periapical surgery, than those whithout a good coronal seal (9). However, Song *et al*. (15) in a retrospective study concluded that the possibility of re-infection of a tooth with a poor coronal seal can be avoided if a good apical seal is obtained.

The proximity of the maxillary sinus is not a contraindication to perform periapical surgery in an upper premolar or molar (13,14). Even though, a study made by Kreisler *et al*. (10) found a significant decrease in success rate in teeth with postoperative oroantral fistula after 6 to 12 months.

A 3 mm apicectomy has to be performed to eliminate all apical ramifications and lateral canals and to avoid reinfection of the periapical area and therefore the recurrence of the lesion (14-16,22). The root section must be perpendicular to the longitudinal axis of the tooth.

Regarding root-end filling materials, the vast majority of the reviewed studies consider the Mineral Trioxide Aggregate (MTA) as an ideal root-end filling material (5-7,9,12,14-16,21,22) as it permits the formation of a physical and biological double seal covering the severed apex is the desired outcome of periapical surgery (14,22).

Numerous studies have made comparisons of the results obtained with the MTA with other filling materials such as IRM, super EBA, glass ionomer or composite (14,15,22). Only one study (15) found no statistically significant difference in success rates between the use of MTA and IRM as retrograde filling material. Two (12,23) of all the included studies in this systematic review obtained higher statistically significant success rates with the use of MTA (91.3% and 86.4%, respectively) compared to Retroplast and SuperEBA.

The flap design should be chosen primarily to facilitate surgical access to the periapical lesion and root and must always follow the basic principles to guarantee adequate vascularization of the flap. A variety of incisions to prepare different types of flaps have been used in periapical surgery. Although there are not studies regarding the influence of the incision in the outcome of periapical surgery, some authors have investigated its effect on periodontal parameters. A study performed by von Arx *et al*. (2) found that the type of incision technique significantly affected changes in the gingival margin during the first year, both at the facial and lingual sites. The sub marginal incision permitted a gain in gingival margin of 0.02 and 0.01 mm at the facial and lingual sites, respectively. On the contrary, the intrasulcular and the papilla-base incisions resulted in a buccal gingival recession of 0.38 and 0.33 mm and in a lingual recession of 0.31 and 0 mm, respectively. Thus, the sub marginal incision is preferred.

Finally, the gingival biotype will modulate the outcome of the soft tissue healing. Patients with a thin gingival biotype are more likely to suffer postoperative gingival recession while those with a thick gum can often develop a periodontal pocket (14). However, no available studies in the literature have proven that gingival biotype is related to the outcome of periapical surgery.

There is an urgent need for high quality prospective studies to be made to increase the scientific evidence on the prognostic factors influencing the long-term success of periapical microsurgery.

## Conclusions

This systematic review reveals that the prognostic factors that affect positively to the outcome of periapical surgery are the following.

- Patient-related factors: patients ≤45 years old.

- Tooth-related factors: upper anterior or premolar teeth, ≤10 sized lesions, non cystic lesions, cases without preoperative signs and symptoms, lesions without periodontal involvement.

- Treatment-related factors: teeth with an adequate root-filling length, MTA as root-end filling material, uniradicular teeth, absence of perforating lesions, apical resection < 3 mm, teeth not associated to an oroantral fistula and teeth with only one periapical surgery.

In function of the scientific quality of the selected articles, a type B recommendation is given in favour of the use of periapical surgery technique in cases that have positive prognostic factors, as cited before.
